# TMT-based quantitative proteomic profiling of human monocyte-derived macrophages and foam cells

**DOI:** 10.1186/s12953-021-00183-x

**Published:** 2022-01-03

**Authors:** Yali Zhang, Yu Fu, Linying Jia, Chenyang Zhang, Wenbin Cao, Naqash Alam, Rong Wang, Weirong Wang, Liang Bai, Sihai Zhao, Enqi Liu

**Affiliations:** 1 Research Institute of Atherosclerotic Disease, Xi’an Jiaotong University Cardiovascular Research Centre, No. 76, Yanta West Road, Xi’an, 710061 Shaanxi China; 2 Laboratory Animal Center, Xi’an Jiaotong University Health Science Centre, Xi’an, 710061 Shaanxi China

**Keywords:** Atherosclerosis, Macrophage, Foam cell, Proteome

## Abstract

**Background:**

Cardiovascular diseases remain the leading cause of morbidity and mortality worldwide, most of which are caused by atherosclerosis. Discerning processes that participate in macrophage-to-foam cell formation are critical for understanding the basic mechanisms underlying atherosclerosis. To explore the molecular mechanisms of foam cell formation, differentially expressed proteins were identified.

**Methods:**

Human peripheral blood mononuclear cells were stimulated with macrophage colony-stimulating factor, and obtained macrophages were transformed into foam cells by oxidized low-density lipoprotein. Tandem mass tag (TMT) labeling combined with mass spectrometry was performed to find associations between foam cell transformation and proteome profiles.

**Results:**

Totally, 5146 quantifiable proteins were identified, among which 1515 and 182 differentially expressed proteins (DEPs) were found in macrophage/monocyte and foam cell/macrophage, respectively. Subcellular localization analysis revealed that downregulated DEPs of macrophages/monocytes were mostly located in the nucleus, whereas upregulated DEPs of foam cells/macrophages were mostly extracellular or located in the plasma membrane. Functional analysis of DEPs demonstrated that cholesterol metabolism-related proteins were upregulated in foam cells, whereas immune response-related proteins were downregulated in foam cells. The protein interaction network showed that the DEPs with the highest interaction scores between macrophages and foam cells were mainly concentrated in lysosomes and the endoplasmic reticulum.

**Conclusions:**

Proteomics analysis suggested that cholesterol metabolism was upregulated, while the immune response was suppressed in foam cells. KEGG enrichment analysis and protein-protein interaction analysis indicated that DEPs located in the endoplasmic reticulum and lysosomes might be key drivers of foam cell formation. These data provide a basis for identifying the potential proteins associated with the molecular mechanism underlying macrophage transformation to foam cells.

**Supplementary Information:**

The online version contains supplementary material available at 10.1186/s12953-021-00183-x.

## Background

Despite extensive efforts to study its pathogenesis and develop effective drugs, atherosclerosis remains the leading cause of mortality and disability worldwide. Atherosclerosis is responsible for coronary artery disease, stroke, and peripheral vascular diseases in the human population [1]. As a progressive disease, atherosclerosis is characterized by the accumulation of lipids and fibrous elements in the arterial intima [2]. Foam cells are key components of atherosclerotic plaque and play an important role in all atherosclerotic lesions, from the earliest fatty streak formations to the most advanced atheromas. In the early stages of atherosclerosis, foam cell accumulation in the arterial wall forms fatty streaks, the first sign of atherosclerosis, which is visible without magnification [3]. In the last phase of atherosclerosis, foam cells can become necrotic and are encapsulated by a thin fibrous cap that can rupture, resulting in thrombosis and vessel occlusion [4].

Macrophages serve as an important source of foam cell formation [5]. Atherosclerosis is initiated by the recruitment of circulating monocytes into the injured vessel intima. Under the stimulation of chemokines and adhesion factors, including P-selectin, VCAM-1(vascular cell adhesion molecule-1), ICAM-1(intercellular cell adhesion molecule-1), MCP-1 (monocyte chemoattractant protein 1), and M-CSF (monocyte colony-stimulating factor) [6], circulating monocytes move into the sub-endothelium of vessel walls and differentiate into macrophages, which subsequently transform into foam cells after engulfing oxidized low-density lipoproteins (ox-LDL) or other modified lipoproteins [7]. Given these important findings, macrophage-derived foam cell formation has emerged as an attractive target for therapeutic intervention and imaging of disease progression. Of note, an inducing factor is necessary for the differentiation of circulating monocytes into macrophages. M-CSF, an important factor in the development, chemotaxis, proliferation, differentiation, and activation of monocytes and macrophages is the most commonly used growth factor for in vitro studies [8, 9]. In vivo, low-density lipoproteins (LDL) are oxidized in atherosclerotic lesions in both humans and transgenic apolipoprotein Ε-deficient mice, whereas plasma LDL is normally not oxidized; the in vitro incubation of macrophages with ox-LDL and not with native LDL led to cholesterol ester accumulation [10–12]. ox-LDL was shown to enhance the uptake of modified lipoproteins via the macrophage scavenger receptor [13]. Therefore, macrophages incubated with ox-LDL to induce foam cells are a common method for studying atherosclerosis in vitro.

In general, the process of macrophage endocytosis of ox-LDL to form foam cells involves cholesterol uptake, esterification, and cholesterol efflux [14]. Macrophages serve as scavenger cells containing scavenger receptors, including CD36 and SR-A, which can recognize and bind to modified lipoproteins for cellular degradation and storage. After ox-LDL endocytosis by macrophages, the cholesterol ester (CE) carried by these particles is hydrolyzed to free cholesterol (FC) in the lysosomes, which is subsequently released into the cytosol. To prevent FC-associated cell toxicity [15], FC in the cytosol is either excluded by ATP-binding cassette transporters, including ABCA1 and ABCG1, or re-esterified by ACAT1 in the endoplasmic reticulum and stored as CE in cytoplasmic lipid droplets (LDs). CE in LDs is hydrolyzed to FC by neutral cholesterol ester hydrolases (nCEHs) or autophagy [16]. Excessive CE accumulates in macrophages, resulting in the formation of foam cells.

Although many proteins have been confirmed to affect the formation of macrophage-derived foam cells, limited information on the overall profiles of differentially expressed proteins (DEPs) expressed during the monocyte-to-macrophage and macrophage-to-foam cell transitions is currently available. Tandem mass tag (TMT)-based proteomics technology enabled more comprehensive and accurate data acquisition, contributing toward elucidating the pathological mechanisms associated with various biological processes. DEPs expressed in response to stimulation may be key molecules affecting pathological processes. Here, we explored the biological processes and potential targets influencing foam cell formation, the main components of atherosclerotic lesions, using in vitro TMT proteomics-based protein profile identification and quantification of human peripheral blood mononuclear cells (PBMCs).

## Methods

### PBMC isolation

Blood samples from healthy male volunteers (age range, 18–21 years) were collected into an EDTA anticoagulant tube. For precision and mass accuracy analyses, blood samples were pooled from 20 individual samples. First, the blood samples were diluted with PBS (1/1 v/v), carefully loaded onto a Ficoll gradient, and centrifuged at 400×g for 30 min at room temperature. PBMCs were collected at the interface and washed three times with PBS (at 400×g for 10 min). Next, monocytes were purified by positive selection using specific monoclonal antibodies, anti-human CD14 magnetic particles (Catalog No. 557769; BD Biosciences, CA, USA) coupled to magnetic beads. Finally, positive cells were resuspended in RPMI-1640 medium (Hyclone, UT, USA) containing penicillin and streptomycin. The study was approved by the Ethics Committee of the Xi’an Jiaotong University Health Science Center (No. 2018–485).

### Cell culture

Monocytes isolated from blood were seeded in cell culture plates at a density of 1 × 10^6^ /mL and grown in RPMI 1640 medium containing 10% fetal serum (v/v), penicillin (100 units/mL), and streptomycin (100 μg/mL) and kept at 37 °C in an atmosphere of 5% CO_2_. To induce macrophages, monocytes were cultured for 7 d in the presence of 100 ng/mL recombinant human macrophage colony-stimulating factor (M-CSF) (Catalog No. 300–25, PeproTech, France). The purity of monocytes and macrophages was evaluated by flow cytometry. For foam cell formation, macrophages were incubated with 50 μg/mL ox-LDL (Yiyuan Bio-technologies, Guangzhou, China) in culture medium for 48 h, and oil red O staining was used to identify whether foam cells were successfully induced.

### Flow cytometry

To detect the efficiency of monocyte transformation into macrophages, monocytes and macrophages were incubated with specific antibodies against CD11b (Catalog No. 561688, BD Biosciences), and positive cells were detected using flow cytometry. In brief, cells were harvested, washed, and the cell suspension was adjusted to a concentration of 1 × 10^6^ cells/mL in ice-cold PBS, 10% fetal calf serum, and 1% sodium azide. We added 1 μg/mL of PE-conjugated CD11b to the cell suspension, which was then incubated for 30 min in the dark at room temperature. The cells were washed three times, centrifuged at 400×g for 5 min, and resuspended in ice-cold PBS, 10% fetal calf serum, and 1% sodium azide. Cells were stored in the dark at 4 °C until analysis.

### Oil red O staining

As foam cells uptake lipids during their formation, oil red O staining was used to identify foam cells. After the culture medium was discarded, cells were washed twice with PBS and fixed with formalin for 10 min. The solution was rinsed with PBS for 1 min and then with 60% isopropanol for 15 s. Cells were then exposed to oil red O for 1 min in the dark at 37 °C, rinsed with 60% isopropanol for 15 s, and then washed three times with PBS for 3 min each. Finally, after being sealed, cells were observed under a Nikon light microscope.

### Protein extraction and digestion

Cell samples were sonicated three times on ice using a high-intensity ultrasonic processor (Scientz) in lysis buffer (8 M urea, 1% Protease Inhibitor Cocktail). The remaining debris was removed by centrifugation at 12,000×g at 4 °C for 10 min. Finally, the supernatant was collected, and the protein concentration was determined using a BCA kit according to the manufacturer’s instructions. For digestion, the protein solution was reduced with 5 mM dithiothreitol for 30 min at 56 °C and alkylated with 11 mM iodoacetamide for 15 min at room temperature in the dark. The protein sample was then diluted by adding 100 mM TEAB to a urea concentration of less than 2 M. Finally, trypsin was added at a 1:50 trypsin-to-protein mass ratio for the first digestion overnight and 1:100 trypsin-to-protein mass ratio for a second 4 h digestion.

### TMT labeling and HPLC fractionation

After trypsin digestion, the peptides were desalted using a Strata X C18 SPE column (Phenomenex, CA, USA) and vacuum-dried. The peptide was reconstituted in 0.5 M TEAB and processed according to the manufacturer’s protocol for the TMT kit. The tryptic peptides were fractionated into 18 fractions by high pH reverse-phase HPLC using Agilent 300 Extend C18 column (5 μm particles, 4.6 mm ID, 250 mm length). Briefly, peptides were separated into 80 fractions with a 2 to 60% acetonitrile gradient in 10 mM ammonium bicarbonate at pH 10, over 80 min. Then, peptides were combined into 18 fractions and dried by vacuum centrifuging. Three replicates per condition were performed. Nine TMT labels (126, 127 N,127C,128 N, 128C, 129 N, 130C, 131) were used per run and 18 TMT runs were performed.

### LC-MS/MS analysis

The tryptic peptides were dissolved in solvent A (an aqueous solution containing 0.1% formic acid and 2% acetonitrile) and directly loaded onto chromatographic column ReproSil-Pur Basic C18 (1.9 μm particles, 100 μm ID, 25 cm length). The gradient ranged from 9 to 26% solvent B (an aqueous solution containing 0.1% formic acid and 90% acetonitrile) over 40 min, 26 to 35% in 14 min, and was increased to 80% in 3 min, then hold at 80% for the last 3 min, all at a constant flow rate of 350 nL/min on an EASY-nLC 1000 UPLC system.

The peptides were subjected to NSI source (the standard source accompanying the Q Exactive TM Plus) followed by tandem mass spectrometry (MS/MS) in Q Exactive™ Plus (Thermo) coupled online to the UPLC. The electrospray voltage applied was 2.1 kV. The m/z scan range was 350 to 1800 for a full scan, and intact peptides were detected in the Orbitrap at a resolution of 70,000. Peptides were then selected for MS/MS using the NCE setting as 28, and the fragments were detected in the Orbitrap at a resolution of 35,000. The data-dependent procedure that we performed alternated between one MS scan followed by 20 MS/MS scans with a 15.0 s dynamic exclusion. Automatic gain control (AGC) was set at 5E4. The fixed first mass was set to 100 m/z.

### Database search

The resulting MS/MS data were processed using the MaxQuant search engine (v.1.5.2.8). Tandem mass spectra were searched against the human SwissProt database (downloaded on 16 August 2018) concatenated with a reverse decoy database. Trypsin/P was used as a cleavage enzyme, allowing up to two missing cleavages. The mass tolerance for precursor ions was set as 20 ppm in the first search and 5 ppm in the main search, and the mass tolerance for fragment ions was set as 0.02 Da. Cys carbamidomethyl was specified as a fixed modification, and Met acetylation and oxidation were specified as variable modifications. FDR was adjusted to < 1%, and the minimum score for modified peptides was set at > 40.

### Bioinformatics analysis

The proteomic results were analyzed using multiple approaches. The Gene Ontology (GO) annotation proteome was derived from the UniProt-GOA database (http://www.ebi.ac.uk/GOA/). Wolfpsort, a subcellular localization prediction, was used to predict subcellular localization. The Kyoto encyclopedia of genes and genomes (KEGG) database was used to annotate protein pathways. First, the KEGG online service tool, KAAS, was used to annotate the protein’s KEGG database description. The annotation results were mapped to the KEGG pathway database using the KEGG online service tool, KEGG mapper. GO annotation and KEGG database were used to identify DEP enrichment by a two-tailed Fisher’s exact test to test the enrichment of the DEPs against all identified proteins. Protein-protein interaction (PPI) network analysis was conducted using STRING version 11.0 (https://string-db.org/). The STRING-generated network was visualized and edited using Cytoscape version 3.8.2.

## Results

### Sample preparation

**Fig. 1:**
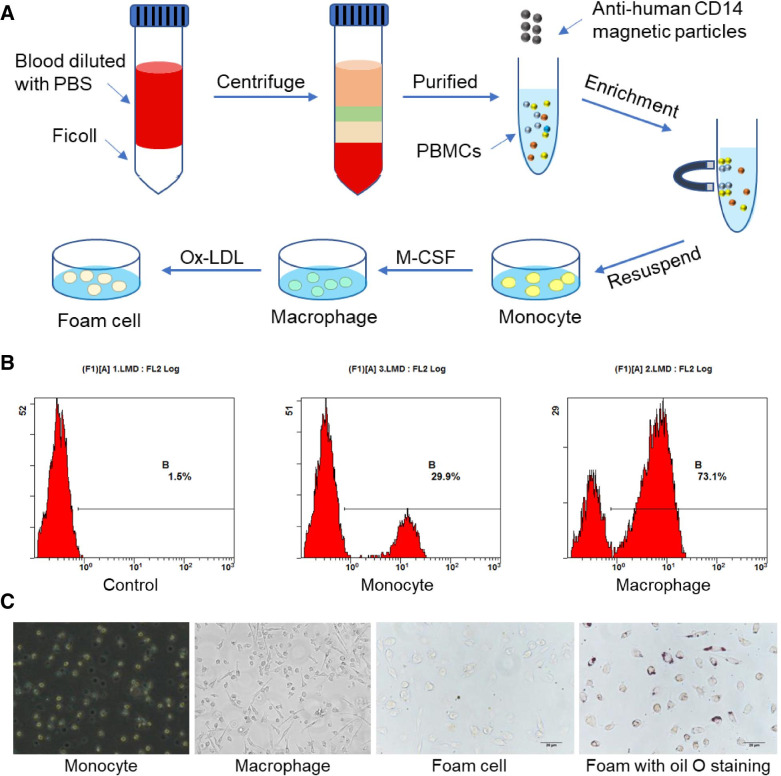
Collection and identification of cell samples. **A** Procedure for collecting human peripheral blood mononuclear cells (PBMCs), monocytes, macrophages, and foam cells. **B** Macrophages were identified with CD11b antibodies using flow cytometry. **C** Representative images of cell morphology of monocytes, macrophages, foam cells, and oil red O stained-foam cells

We isolated PBMCs from whole blood and cultured them in vitro to obtain monocytes, macrophages, and foam cells induced by M-CSF and ox-LDL; the sampling procedure is shown in Fig. 1A. Before testing by mass spectrometry, monocytes were purified with CD14 microbeads as CD14 is a monocyte surface marker, and macrophages were identified by flow cytometry using CD11b antibodies. The results showed that the CD11b + population increased from 29.9 to 73.1% after M-CSF induction (Fig. 1B). Moreover, macrophages in samples were adherent cells. Macrophages phagocytose lipids to form foam cells. The results showed that ox-LDL-induced cells, stained with oil red O, presented red lipid droplets (Fig. 1C).

### Overview of protein identification on TMT technology

**Fig. 2:**
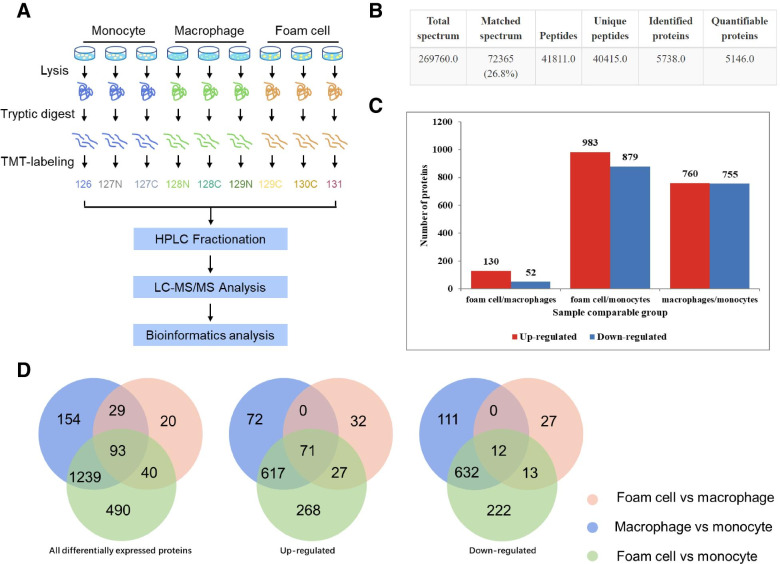
Overview of differentially expressed proteins (DEPs). **A** Schematic illustration of the proteomic analytical steps. **B** Summary of qualitative data identified in monocytes, macrophages, and foam cells. **C** Number of DEPs in foam cell/macrophage, foam cell/monocyte, and macrophage/monocyte. **D** Venn diagram showing the overlap in foam cell/macrophage, foam cell/monocyte, and macrophage/monocyte

Total proteins obtained from monocytes, macrophages, and foam cells were separately analyzed for LC-MS/MS identification with three replicates (Fig. 2A). Quality control was performed to check the MS data. Results indicated that the MS data satisfied the subsequent advanced analysis (see Additional file 1). We uploaded raw data to ProteomeXchange via the PRIDE database (Project accession: PXD028363). Altogether, 5738 proteins were identified; of these, 5146 proteins were quantified (Fig. 2B, Additional file 2). Compared with foam cells/macrophages, the foam cells/monocytes and macrophages/monocytes had more DEPs at the same differential multiple (Additional file 3). To control the number of proteins analyzed by bioinformatics within the appropriate range, we defined DEPs as proteins expressed when foam cells/monocytes, macrophages/monocytes, or foam cells/macrophages had a fold change > 1.5 or < 0.67 and *p* < 0.05 (*p*-value was calculated by the two-sample two-tailed T-test method). According to this definition, there were 1515 (760 upregulated and 755 downregulated), 182 (130 upregulated and 52 downregulated), and 1862 (983 upregulated and 879 downregulated) DEPs in macrophages/monocytes, foam cells/macrophages, and foam cells/monocytes, respectively (Fig. 2C). Figure 2D shows the number of DEPs shared by the three groups.

### Subcellular localization of DEPs

**Fig. 3:**
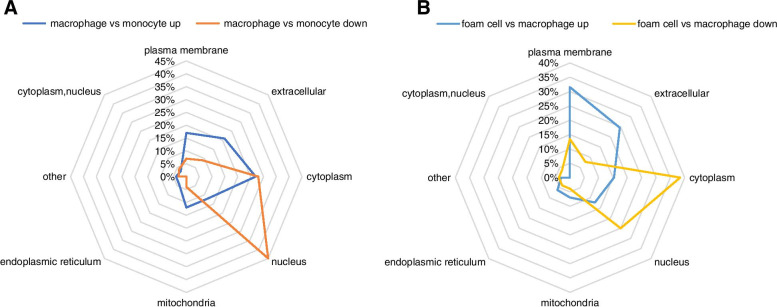
Subcellular location of differentially expressed proteins (DEPs). **A** Subcellular location of DEPs in monocytes and macrophages. **B** Subcellular location of DEPs in foam cells and macrophages

Subcellular localization is the main determinant of protein function. Here, we used Wolfpsort, a subcellular localization prediction software, to predict the subcellular localization of DEPs. Subcellular localization showed that upregulated DEPs in macrophages/monocytes were mainly found in the cytoplasm, followed by the extracellular and plasma membrane. In contrast, downregulated DEPs were mainly found in the nucleus (Fig. 3A). Upregulated DEPs in foam cells/macrophages were primarily found in the cell membrane and extracellular matrix and downregulated DEPs primarily occupied the cytoplasm and nucleus (Fig. 3B).

### Functional enrichment analysis of DEPs

**Fig. 4:**
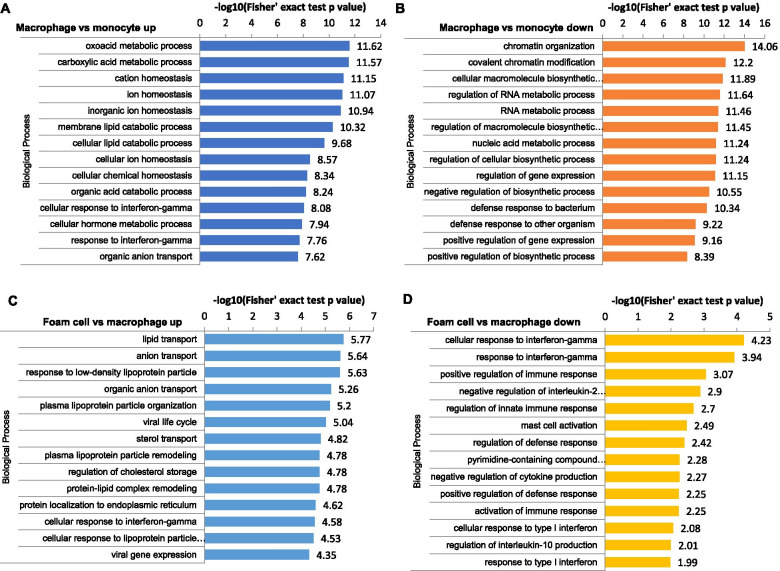
Gene Ontology (GO)-based functional enrichment analysis of differentially expressed proteins (DEPs). GO-based functional enrichment analysis of DEPs in the macrophage/monocyte (**A, B**); GO-based functional enrichment analysis of DEPs in the foam cell/macrophage (**C, D**)

To better investigate the biological function of DEPs, we performed a comparative analysis based on GO enrichment and the KEGG pathway enrichment. A Fisher’s exact test *p*-value was obtained to evaluate the enrichment analysis with the quantified proteins as references. As shown in Fig. 4, during the monocyte-to-macrophage transformation, upregulated DEPs were enriched in the biological processes related to immune responses, and downregulated DEPs were enriched in the biological processes related to chromatin. For DEPs of foam cells/macrophages, results indicated that upregulated proteins were enriched in the biological process of lipid transport and response to LDL particles. In contrast, downregulated proteins were involved with cellular response to interferon-gamma and positive regulation of immune response.

**Fig. 5:**
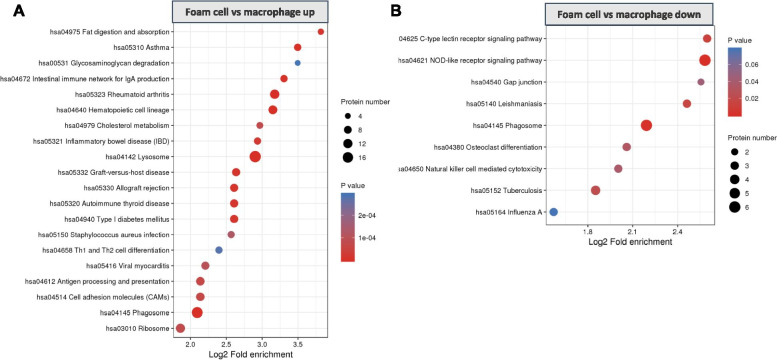
KEGG pathway enrichment analysis of differentially expressed proteins in foam cell/macrophage

The KEGG pathway enrichment analysis indicated that upregulated DEPs of macrophages/monocytes were significantly enriched in antigen processing and presentation, lysosome, and phagocytosis pathways; the downregulated proteins were enriched in DNA replication (Additional file 4). For foam cells/macrophages, the KEGG pathway enrichment analysis showed that proteins involved in cholesterol metabolism and lysosome were upregulated in foam cells/macrophages. In contrast, proteins in the NOD-like receptor signaling pathway and the C-type lectin receptor signaling pathway were significantly downregulated (Fig. 5).

### Interaction network of DEPs

**Fig. 6:**
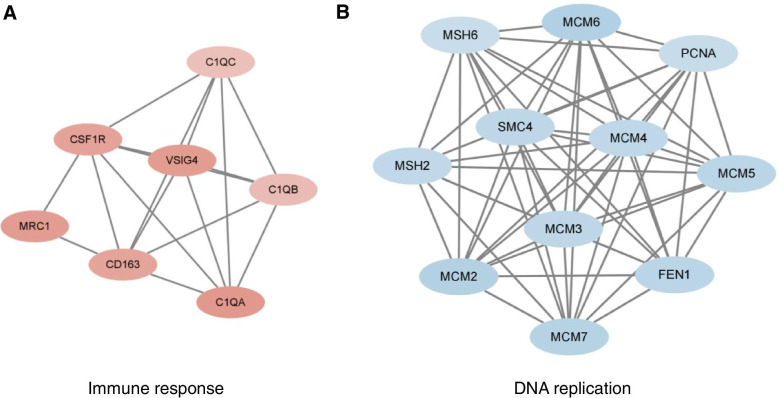
Top two modules from the protein-protein interaction network according to the Molecular Complex Detection (MCODE) score in macrophage/monocyte. **A**, the upregulated proteins in macrophages; **B**, the downregulated proteins in macrophages

To better comprehend the interactions between the DEPs, STRING analysis combined with Cytoscape software was used to visualize the PPI networks. Notably, PPI analysis of DEPs in macrophages/monocytes was performed on the proteins with fold change > 2, because the number of proteins with fold change > 1.5 was too large. The PPI analysis indicated extensive interactions among DEPs of macrophages/monocytes. Molecular Complex Detection (MCODE), a graph-theoretic clustering algorithm, captured the modules with strong interacting proteins. As shown in Fig. 6A, interactions among upregulated proteins from the macrophages/monocytes were mostly immune response-related, whereas downregulated proteins were related to DNA replication. Furthermore, we performed a PPI network analysis on selected GO terms, including receptor-mediated endocytosis, immune effector process, and ion transport. The identified nodes with high interaction scores that might be potential targets for future research are listed in Additional file 5.

### DEPs located in the lysosome and endoplasmic reticulum

**Fig. 7:**
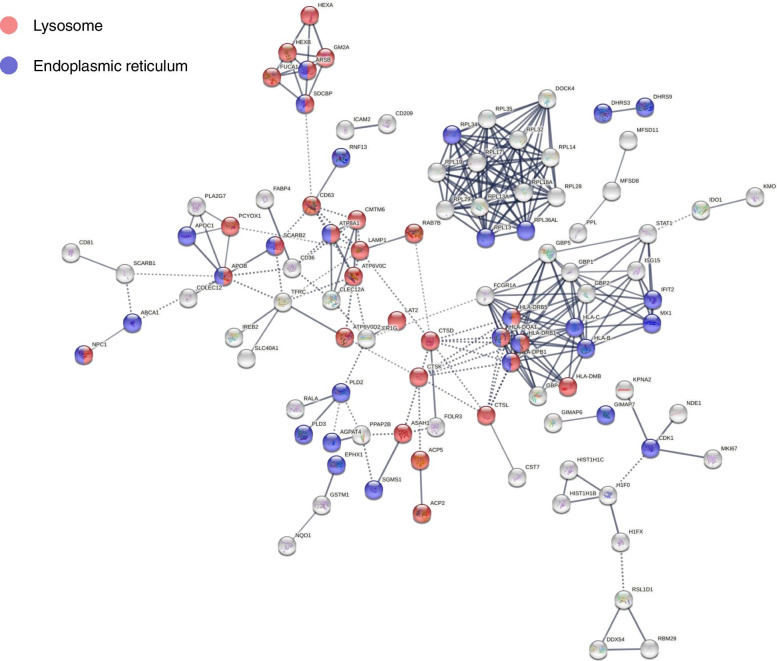
Interaction network of foam cells/macrophages differentially expressed proteins located in lysosomes and endoplasmic reticulum

According to GO analysis, in addition to lysosomes, the proteins located in the endoplasmic reticulum were also widely enriched in the top 50 proteins with the strongest interaction in the foam cell/macrophage group (Additional file 6). Next, we mapped the distribution of all foam cell/macrophage DEPs in the lysosomes and endoplasmic reticulum in the interaction network (Fig. 7). In total, of the 182 DEPs, 38 were located in the lysosome (see Table 1), and 40 were located in the endoplasmic reticulum (see Table 2).

**Table 1:** List of differentially expressed proteins (foam cell/macrophage) located in the lysosome

Protein accession	Protein description	Gene name	Fold change
P13761	HLA class II histocompatibility antigen, DRB1-7 beta chain	HLA-DRB1	11.602
P04114	Apolipoprotein B-100	APOB	7.521
Q8N8Y2	V-type proton ATPase subunit d 2	ATP6V0D2	2.817
P13686	Tartrate-resistant acid phosphatase type 5	ACP5	2.518
P04066	Tissue alpha-L-fucosidase	FUCA1	2.247
Q95IE3	HLA class II histocompatibility antigen, DRB1-12 beta chain	HLA-DRB1	2.234
P20039	HLA class II histocompatibility antigen, DRB1-11 beta chain	HLA-DRB1	2.107
Q86WA9	Sodium-independent sulfate anion transporter	SLC26A11	2.067
P11279	Lysosome-associated membrane glycoprotein 1	LAMP1	2.002
Q9NX76	CKLF-like MARVEL transmembrane domain-containing protein 6	CMTM6	1.985
P43235	Cathepsin K	CTSK	1.884
P01909	HLA class II histocompatibility antigen, DQ alpha 1 chain	HLA-DQA1	1.882
P27449	V-type proton ATPase 16 kDa proteolipid subunit	ATP6V0C	1.87
Q14108	Lysosome membrane protein 2	SCARB2	1.844
O15118	NPC intracellular cholesterol transporter 1	NPC1	1.786
P08962	CD63 antigen	CD63	1.76
Q8N357	Solute carrier family 35 member F6	SLC35F6	1.748
P11117	Lysosomal acid phosphatase	ACP2	1.705
P07339	Cathepsin D	CTSD	1.681
Q96AH8	Ras-related protein Rab-7b	RAB7B	1.677
P07686	Beta-hexosaminidase subunit beta	HEXB	1.674
P28068	HLA class II histocompatibility antigen, DM beta chain	HLA-DMB	1.67
P17900	Ganglioside GM2 activator	GM2A	1.619
Q96NW4	Ankyrin repeat domain-containing protein 27	ANKRD27	1.619
P15848	Arylsulfatase B	ARSB	1.597
Q30154	HLA class II histocompatibility antigen, DR beta 5 chain	HLA-DRB5	1.582
P07711	Cathepsin L1	CTSL	1.574
Q8IY95	Transmembrane protein 192	TMEM192	1.566
O00115	Deoxyribonuclease-2-alpha	DNASE2	1.564
Q9Y2Q0	Phospholipid-transporting ATPase IA	ATP8A1	1.553
Q9UHG3	Prenylcysteine oxidase 1	PCYOX1	1.539
Q13510	Acid ceramidase	ASAH1	1.538
P06865	Beta-hexosaminidase subunit alpha	HEXA	1.518
P04440	HLA class II histocompatibility antigen, DP beta 1 chain	HLA-DPB1	1.517
O00560	Syntenin-1	SDCBP	1.51
P51688	N-sulphoglucosamine sulphohydrolase	SGSH	1.505
Q9GZY6	Linker for activation of T-cells family member 2	LAT2	0.572
A6NI72	Putative neutrophil cytosol factor 1B	NCF1B	0.53

**Table 2:** List of DEPs (foam cell/macrophage) located in the endoplasmic reticulum

Protein accession	Protein description	Gene name	Fold change
P13761	HLA class II histocompatibility antigen, DRB1-7 beta chain	HLA-DRB1	11.602
P04114	Apolipoprotein B-100	APOB	7.521
P05090	Apolipoprotein D	APOD	5.59
Q99541	Perilipin-2	PLIN2	4.709
P02654	Apolipoprotein C-I	APOC1	2.729
O95477	ATP-binding cassette sub-family A member 1	ABCA1	2.568
Q95IE3	HLA class II histocompatibility antigen, DRB1-12 beta chain	HLA-DRB1	2.234
P20039	HLA class II histocompatibility antigen, DRB1-11 beta chain	HLA-DRB1	2.107
Q86WA9	Sodium-independent sulfate anion transporter	SLC26A11	2.067
Q9BPW9	Dehydrogenase/reductase SDR family member 9	DHRS9	2.032
Q9H2F3	3 beta-hydroxysteroid dehydrogenase type 7	HSD3B7	1.992
O75911	Short-chain dehydrogenase/reductase 3	DHRS3	1.966
P30499	HLA class I histocompatibility antigen, Cw-1 alpha chain	HLA-C	1.909
P01909	HLA class II histocompatibility antigen, DQ alpha 1 chain	HLA-DQA1	1.882
Q14108	Lysosome membrane protein 2	SCARB2	1.844
Q95365	HLA class I histocompatibility antigen, B-38 alpha chain	HLA-B	1.815
O14939	Phospholipase D2	PLD2	1.79
O15118	NPC intracellular cholesterol transporter 1	NPC1	1.786
P20591	Interferon-induced GTP-binding protein Mx1	MX1	1.776
Q9NRZ5	1-acyl-sn-glycerol-3-phosphate acyltransferase delta	AGPAT4	1.688
O43567	E3 ubiquitin-protein ligase RNF13	RNF13	1.683
P49207	60S ribosomal protein L34	RPL34	1.653
Q96NN9	Apoptosis-inducing factor 3	AIFM3	1.636
P07099	Epoxide hydrolase 1	EPHX1	1.615
Q969Q0	60S ribosomal protein L36a-like	RPL36AL	1.604
P15848	Arylsulfatase B	ARSB	1.597
O00767	Acyl-CoA desaturase	SCD	1.594
Q30154	HLA class II histocompatibility antigen, DR beta 5 chain	HLA-DRB5	1.582
P09913	Interferon-induced protein with tetratricopeptide repeats 2	IFIT2	1.575
Q8IV08	Phospholipase D3	PLD3	1.564
Q9Y2Q0	Phospholipid-transporting ATPase IA	ATP8A1	1.553
P04440	HLA class II histocompatibility antigen, DP beta 1 chain	HLA-DPB1	1.517
P26373	60S ribosomal protein L13	RPL13	1.513
O00560	Syntenin-1	SDCBP	1.51
Q16850	Lanosterol 14-alpha demethylase	CYP51A1	0.594
Q86VZ5	Phosphatidylcholine:ceramide cholinephosphotransferase 1	SGMS1	0.583
P30504	HLA class I histocompatibility antigen, Cw-4 alpha chain	HLA-C	0.536
A6NI72	Putative neutrophil cytosol factor 1B	NCF1B	0.53
Q8NHV1	GTPase IMAP family member 7	GIMAP7	0.513
P06493	Cyclin-dependent kinase 1	CDK1	0.366

## Discussion

Monocytes and macrophages are key cells in the initiation and progression of atherosclerosis. When blood vessels are damaged, monocytes in the blood are recruited to the subcutaneous vessels under the action of cytokines to form macrophages, which phagocytose lipids and form foam cells; this is the basic process of the formation of atherosclerotic lesions [17]. In addition, macrophages can affect the progression of atherosclerosis through inflammation [18]. Several studies have demonstrated that protein expression changes in macrophages affect atherosclerosis, such as CD36 and FABP [19, 20]. Therefore, it is plausible to consider macrophages as targets to identify potential therapeutic targets to treat or prevent atherosclerosis. Several studies have used proteomics to identify DEPs in monocytes, macrophages, and foam cells; however, due to technical limitations, the number of identified proteins is extremely limited, insufficient to comprehensively display the changes in protein expression during the process of cell transformation that characterizes atherosclerosis [21–24]. In this study, we performed protein quantification based on TMT analysis of human PBMCs, monocyte-derived macrophages induced by M-CSF, and foam cells formed from macrophages exposed to oxidized low-density lipoprotein. Through the functional enrichment and network interaction analysis of DEPs, we provided extensive molecular profiling to analyze the molecular network and highlighted changes in the expression of proteins in the lysosome and endoplasmic reticulum after macrophage phagocytosis of ox-LDL.

In our study, downregulated DEPs of macrophages/monocytes were significantly enriched in the cell cycle (Additional file 4), suggesting that monocyte differentiation into macrophages significantly altered the proliferative ability of monocytes, favoring an inverse relationship between cellular proliferation and differentiation. This finding is consistent with the previous understanding that considers macrophages as terminally differentiated immune cells that develop from monocytes and are unable to re-enter the cell cycle [25]. Moreover, KEGG pathway enrichment showed that, in macrophages, phagocytosis, antigen processing, and presentation were enhanced compared to monocytes. It is well known that phagocytosis and adaptive immunity are critical features of macrophages. These results support the reliability of our high-throughput proteomic data.

Atherosclerosis is a chronic inflammatory disease of the vessel wall, primarily driven by an innate immune response through myeloid cells, such as monocytes and macrophages. It is clear that the adaptive immune system in atherosclerosis can be pro- or anti-inflammatory, and thus pro- or anti-atherogenic [26]. It is generally believed that cholesterol accumulation induces macrophages to undergo inflammatory responses. However, our results suggest that the immune response was weakened in lipid-loaded macrophages, as downregulated DEPs were enriched in the C-type lectin receptor and the NOD-like receptor signaling pathways (Fig. 5), involved in the innate immune response and inflammatory activation [27, 28]. These results are consistent with the point revealed from single-cell RNA sequencing analysis of CD45^+^ leukocytes from the murine atherosclerotic aorta, showing that lipid-loaded macrophages are not likely to drive lesion inflammation [29]. Transcriptomic analysis by Spann et al. also demonstrated that foam cell formation was associated with suppression, rather than activation, of inflammatory gene expression [30], and our results support this discovery at the protein level.

Foam cell formation involves the disruption of normal macrophage cholesterol metabolism, which is governed by a homeostatic mechanism that controls the uptake, intracellular metabolism, and efflux of cholesterol [31]. As anticipated, the proteins related to cholesterol metabolism, including CD36, MSR1, LIPA, NPC1, and ABCA1, were upregulated in foam cells compared to macrophages (Additional file 3).

As the sites of cholesterol hydrolysis and esterification, lysosomes and the endoplasmic reticulum are pivotal in regulating lipid metabolism. Our results showed that strong protein-protein interactions were enriched in lysosomes and the endoplasmic reticulum. Several DEPs (Additional file 3), such as LAMP1 [32], CTSK [33], PLD2 [34], PLIN2 [35], identified in lysosomes and endoplasmic reticulum have been shown to affect foam cell formation. Recently, the impact of macrophage autophagy on cholesterol efflux has drawn increasing attention. Cholesterol efflux from macrophages is the first and potentially most important step in reverse cholesterol transport, a process especially relevant to atherosclerosis and the regression of atherosclerotic plaques [14]. LDs are delivered to lysosomes via autophagy, where lysosomal acid lipase hydrolyzes LD/CE to generate FC, usually for ABCA1-dependent efflux; this process is specifically induced upon macrophage cholesterol loading [16]. These findings suggest that attention to the endoplasmic reticulum and lysosomal proteins may provide potential targets for reducing foam cell formation and, thus, ameliorating atherosclerosis.

## Conclusions

To the best of our knowledge, our study provides the most comprehensive comparative proteomics data of monocytes, macrophages, and foam cells in early atherosclerosis. The results showed that cholesterol metabolism was upregulated in foam cells, while the immune response was downregulated in foam cells compared with macrophages. Moreover, functional enrichment analysis and protein network interaction analysis revealed the pivotal role of lysosomes and endoplasmic reticulum in macrophage cholesterol metabolism. Further research of DEPs during foam cell formation will help us gain deeper insights into the pathophysiology of atherosclerosis and develop novel therapeutic alternatives.

## Supplementary Information

**Additional file 1.** The correlation and coefficient of variation for biological replicates. (A) Coefficient of variation. (B) Pearson correlation coefficient of three replications.

**Additional file 2.** List of MS data and proteins identified in monocyte, macrophage, and foam cell by TMT- based quantitative proteomics.

**Additional file 3.** List of differentially expressed proteins (fold change > 1.5, *p* < 0.05) in macrophage/monocyte, foam cell/ macrophage, and foam cell/monocyte.

**Additional file 4.** Kyoto encyclopedia of genes and genomes (KEGG) enrichment analysis of differentially expressed proteins in macrophage/ monocyte.

**Additional file 5.** High interaction nodes obtained by protein-protein interaction (PPI) analysis of Gene Ontology (GO)-based functional enrichment terms in the foam cell/macrophage group.

**Additional file 6.** The differentially expressed proteins located in the endoplasmic reticulum or lysosome with the top 50 strongest interactions.

## Data Availability

All data generated or analyzed during this study are included in this published article and its supplementary information files.

## Abbreviations

CE: Cholesterol ester
DEPs: Differentially expressed proteins
FC: Free cholesterol
GO: Gene Ontology
KEGG: Kyoto encyclopedia of genes and genomes
LDL: Low-density lipoproteins
LDs: Lipid droplets
M-CSF: Monocyte colony-stimulating factor
MS: Mass spectrometry
ox-LDL: Oxidized low-density lipoproteins
PBMCs: Peripheral blood mononuclear cells
PPI: Protein-protein interaction
TMT: Tandem mass tag

## References

[1] Benjamin EJ, Muntner P, Alonso A, Bittencourt MS, Callaway CW, Carson AP (2019). Heart disease and stroke statistics-2019 update: a report from the American Heart Association. Circulation..

[2] Lusis AJ (2000). Atherosclerosis. Nature..

[3] Stary HC, Chandler AB, Glagov S, Guyton JR, Insull W, Rosenfeld ME (1994). A definition of initial, fatty streak, and intermediate lesions of atherosclerosis. A report from the committee on vascular lesions of the council on arteriosclerosis, American Heart Association. Circulation..

[4] Otsuka F, Yasuda S, Noguchi T, Ishibashi-Ueda H (2016). Pathology of coronary atherosclerosis and thrombosis. Cardiovasc Diagn Ther.

[5] Maguire EM, Pearce SWA, Xiao Q (2019). Foam cell formation: a new target for fighting atherosclerosis and cardiovascular disease. Vasc Pharmacol.

[6] Mestas J, Ley K (2008). Monocyte-endothelial cell interactions in the development of atherosclerosis. Trends Cardiovasc Med.

[7] Li AC, Glass CK (2002). The macrophage foam cell as a target for therapeutic intervention. Nat Med.

[8] Jin X, Kruth HS (2016). Culture of macrophage colony-stimulating factor differentiated human monocyte-derived macrophages. J Vis Exp.

[9] Chitu V, Yeung YG, Yu W, Nandi S, Stanley ER (2011). Measurement of macrophage growth and differentiation. Curr Protoc Immunol.

[10] Steinberg D, Parthasarthy S, Carew TE, Khoo JC, Witztum JL (1989). Beyond cholesterol. Modifications of low-density lipoprotein that increase its atherogenicity. N Engl J Med.

[11] Parthasarathy S, Quinn MT, Steinberg D (1988). Is oxidized low density lipoprotein involved in the recruitment and retention of monocyte/macrophages in the artery wall during the initiation of atherosclerosis?. Basic Life Sci.

[12] Quinn MT, Parthasarathy S, Fong LG, Steinberg D (1987). Oxidatively modified low density lipoproteins: a potential role in recruitment and retention of monocyte/macrophages during atherogenesis. Proc Natl Acad Sci U S A.

[13] Kunjathoor VV, Febbraio M, Podrez EA, Moore KJ, Andersson L, Koehn S (2002). Scavenger receptors class A-I/II and CD36 are the principal receptors responsible for the uptake of modified low density lipoprotein leading to lipid loading in macrophages. J Biol Chem.

[14] Ouimet M, Marcel YL (2012). Regulation of lipid droplet cholesterol efflux from macrophage foam cells. Arterioscler Thromb Vasc Biol.

[15] Maxfield FR, van Meer G (2010). Cholesterol, the central lipid of mammalian cells. Curr Opin Cell Biol.

[16] Ouimet M, Franklin V, Mak E, Liao X, Tabas I, Marcel YL (2011). Autophagy regulates cholesterol efflux from macrophage foam cells via lysosomal acid lipase. Cell Metab.

[17] Bobryshev YV (2006). Monocyte recruitment and foam cell formation in atherosclerosis. Micron..

[18] Moore KJ, Sheedy FJ, Fisher EA (2013). Macrophages in atherosclerosis: a dynamic balance. Nat Rev Immunol.

[19] Febbraio M, Guy E, Silverstein RL (2004). Stem cell transplantation reveals that absence of macrophage CD36 is protective against atherosclerosis. Arterioscler Thromb Vasc Biol.

[20] Makowski L, Boord JB, Maeda K, Babaev VR, Uysal KT, Morgan MA (2001). Lack of macrophage fatty-acid-binding protein aP2 protects mice deficient in apolipoprotein E against atherosclerosis. Nat Med.

[21] Kang JH, Ryu HS, Kim HT, Lee SJ, Choi UK, Park YB (2009). Proteomic analysis of human macrophages exposed to hypochlorite-oxidized low-density lipoprotein. Biochim Biophys Acta.

[22] Kang JH, Kim HT, Choi MS, Lee WH, Huh TL, Park YB (2006). Proteome analysis of human monocytic THP-1 cells primed with oxidized low-density lipoproteins. Proteomics..

[23] Fach EM, Garulacan LA, Gao J, Xiao Q, Storm SM, Dubaquie YP (2004). In vitro biomarker discovery for atherosclerosis by proteomics. Mol Cell Proteomics.

[24] Castagna A, Polati R, Bossi AM, Girelli D (2012). Monocyte/macrophage proteomics: recent findings and biomedical applications. Expert Rev Proteomics.

[25] Röszer T. Understanding the Biology of Self-Renewing Macrophages. Cells. 2018;7:103. 10.3390/cells7080103 PMC6115929 30096862

[26] Wolf D, Ley K (2019). Immunity and inflammation in atherosclerosis. Circ Res.

[27] Saxena M, Yeretssian G (2014). NOD-like receptors: master regulators of inflammation and cancer. Front Immunol.

[28] Kingeter LM, Lin X (2012). C-type lectin receptor-induced NF-κB activation in innate immune and inflammatory responses. Cell Mol Immunol.

[29] Kim K, Shim D, Lee JS, Zaitsev K, Williams JW, Kim KW (2018). Transcriptome analysis reveals nonfoamy rather than foamy plaque macrophages are proinflammatory in atherosclerotic murine models. Circ Res.

[30] Spann NJ, Garmire LX, McDonald JG, Myers DS, Milne SB, Shibata N (2012). Regulated accumulation of desmosterol integrates macrophage lipid metabolism and inflammatory responses. Cell..

[31] McLaren JE, Michael DR, Ashlin TG, Ramji DP (2011). Cytokines, macrophage lipid metabolism and foam cells: implications for cardiovascular disease therapy. Prog Lipid Res.

[32] Qiao L, Wang HF, Xiang L, Ma J, Zhu Q, Xu D (2020). Deficient chaperone-mediated autophagy promotes lipid accumulation in macrophage. J Cardiovasc Transl Res.

[33] Lutgens E, Kutgens SPM, Faber BCG, Heeneman S, Gijbels MMJ, De Winther MPJ (2006). Disruption of the cathepsin K gene reduces atherosclerosis progression and induces plaque fibrosis but accelerates macrophage foam cell formation. Circulation..

[34] Ganesan R, Henkels KM, Wrenshall LE, Kanaho Y, Paolo GD, Frohman MA (2018). Oxidized LDL phagocytosis during foam cell formation in atherosclerotic plaques relies on a PLD2-CD36 functional interdependence. J Leukoc Biol.

[35] Paul A, Chang BHJ, Li L, Yechoor VK, Chan L (2008). Deficiency of adipose differentiation-related protein impairs foam cell formation and protects against atherosclerosis. Circ Res.

