# Supplementary figures and images for: TMT-based quantitative proteomic profiling of human monocyte-derived macrophages and foam cells
Source: Proteome Sci. 2022 Jan 3;20:1. doi: 10.1186/s12953-021-00183-x (PMC8725474; doi:10.1186/s12953-021-00183-x)

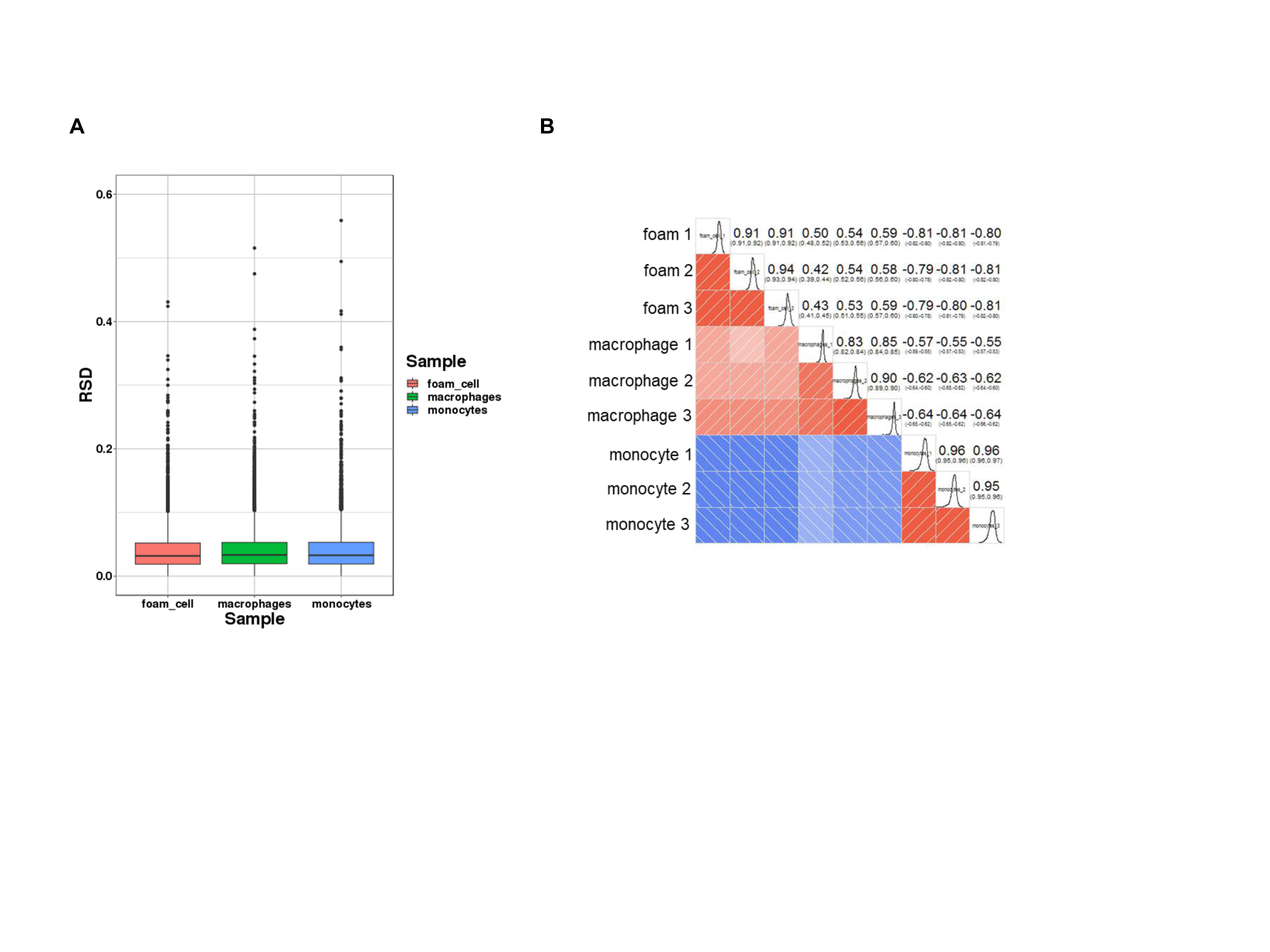

Supplement: Supplementary file 1 — Additional file 1. The correlation and coefficient of variation for biological replicates. (A) Coefficient of variation. (B) Pearson correlation coefficient of three replications. [file 12953_2021_183_MOESM1_ESM.tif]

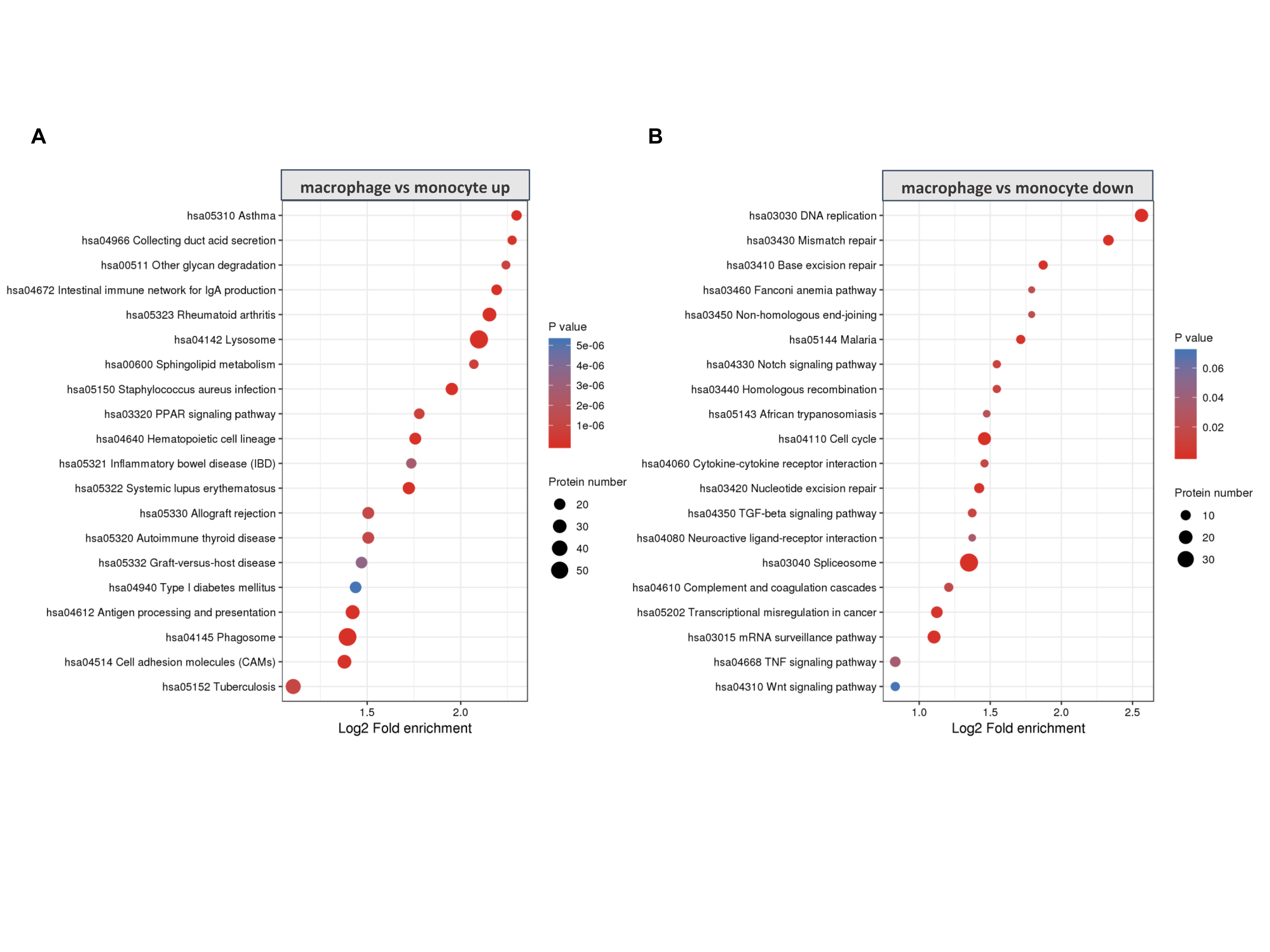

Supplement: Supplementary file 4 — Additional file 4. Kyoto encyclopedia of genes and genomes (KEGG) enrichment analysis of differentially expressed proteins in macrophage/ monocyte. [file 12953_2021_183_MOESM4_ESM.tif]

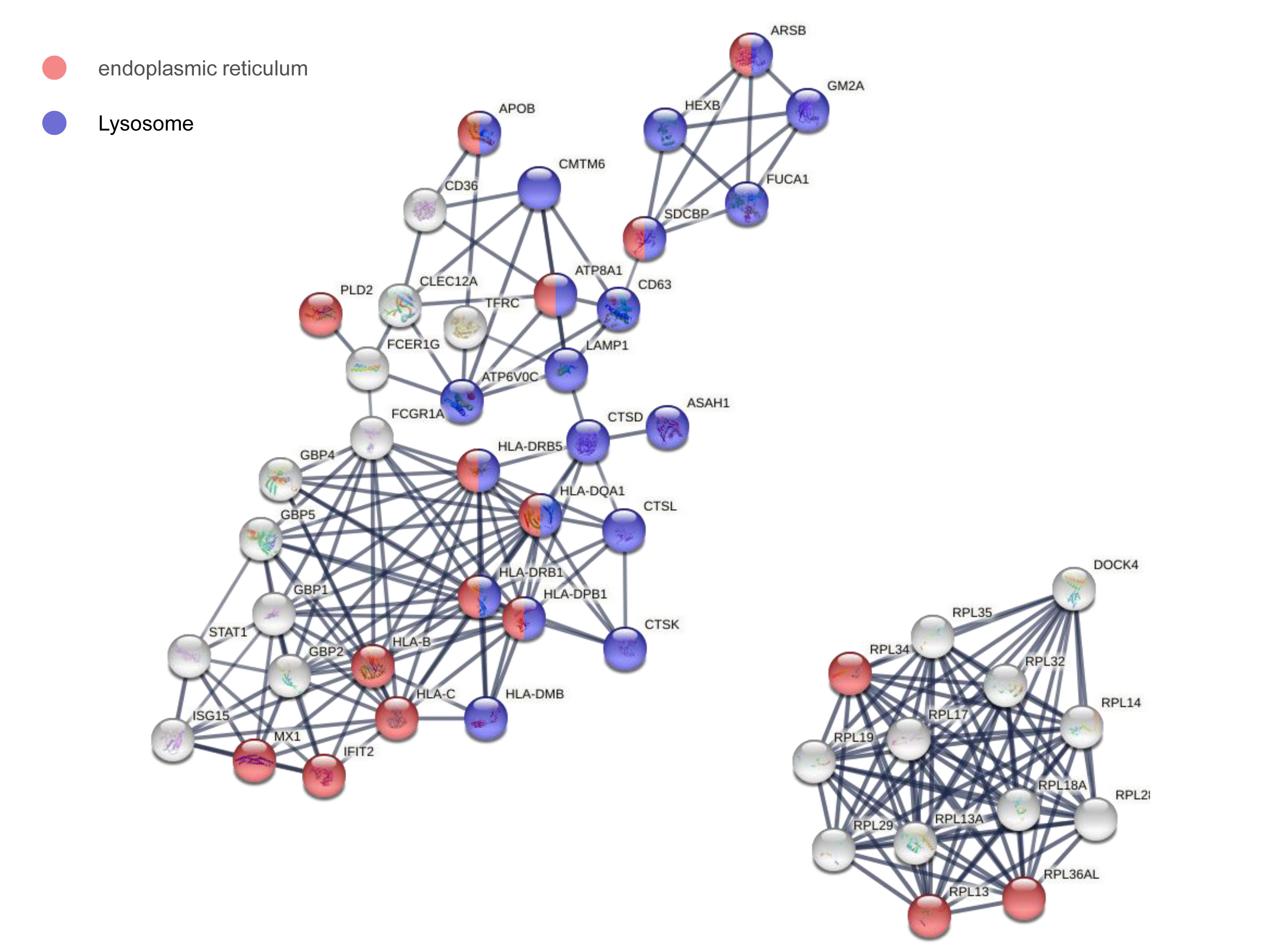

Supplement: Supplementary file 6 — Additional file 6. The differentially expressed proteins located in the endoplasmic reticulum or lysosome with the top 50 strongest interactions. [file 12953_2021_183_MOESM6_ESM.tif]
